# Comparison of Diagnostic Performance Between CT and MRI for Detection of Cartilage Invasion and Tumor Staging in Patients with Laryngeal Cancer: A Systematic Review and Meta-Analysis

**DOI:** 10.3390/cancers18040583

**Published:** 2026-02-10

**Authors:** Ingrid-Denisa Barcan, Dan Ionel Orbulescu, Andreea-Mihaela Banța, Alexandru Catalin Motofelea, Dana Emilia Movila, Razvan Gheorghe Diaconescu, Emanuela Stan, Eugen Radu Boia, Delia Ioana Horhat

**Affiliations:** 1 Department of Doctoral Studies, “Victor Babes” University of Medicine and Pharmacy Timisoara, Eftimie Murgu Square No. 2, 300041 Timisoara, Romania; ingrid.barcan@umft.ro (I.-D.B.); andreea.banta@umft.ro (A.-M.B.); alexandru.motofelea@umft.ro (A.C.M.); 2 ENT Department, University of Medicine and Pharmacy “Victor Babes”, 300041 Timisoara, Romania; 3 ENT Department, Emergency City Hospital, 300254 Timisoara, Romania; 4 Plastic Surgery Department, “Victor Babes” University of Medicine and Pharmacy, 300041 Timisoara, Romania; 5 Centre for Molecular Research in Nephrology and Vascular Disease/MOL-NEPHRO-VASC, “Victor Babes” University of Medicine and Pharmacy Timisoara, 300041 Timisoara, Romania; 6 Department VI—Cardiology, University Clinic of Internal Medicine and Ambulatory Care, Prevention and Cardiovascular Recovery, “Victor Babes” University of Medicine and Pharmacy, 300041 Timisoara, Romania; man.dana@umft.ro; 7 Department of Oncology, OncoHelp Hospital Timisoara, Ciprian Porumbescu Street, No. 59, 300239 Timisoara, Romania; razvan.diaconescu@umft.ro; 8 Department of Neuroscience, Discipline of Forensic Medicine, Bioethics, Deontology and Medical Law, “Victor Babes” University of Medicine and Pharmacy, 300041 Timisoara, Romania

**Keywords:** laryngeal cancer, computed tomography, magnetic resonance imaging, cartilage invasion, tumor staging, diagnostic accuracy meta-analysis

## Abstract

Background: Laryngeal cancer constitutes a major burden. Accurate staging is important to determine the optimal treatment approach. However, a head-to-head comparison of diagnostic performance between CT and MRI in patients with laryngeal cancer is lacking. Furthermore, the performance of CT and MRI in detecting the invasion of different laryngeal structures is yet to be determined. Objective: To compare the diagnostic performance of CT and MRI in patients with laryngeal cancer. Methods: We searched PubMed, Scopus, and Web of Science in November 2025 for cohort studies that compared both index tests (CT and MRI) to histopathological examination of laryngeal cancer. We assessed the quality of the included studies using the QUADAS 2 tool. A bivariate meta-analysis was performed using R and RevMan software to compare pooled sensitivity and specificity, with summary receiver operating characteristic (SROC) curves generated for each outcome. Results: We included eight studies. The pooled data showed that CT was significantly less sensitive compared to MRI in detecting invasion of the thyroid cartilage with an absolute difference of −0.43 (95% CI −0.59 to −0.26), cricoid cartilage −0.65 (95% CI −1.14 to −0.15), paraglottic space −0.5 (95% CI −0.68 to −0.32), and anterior commissure −0.48 (95% CI −0.1 to −0.86). Specificity was similarly high for both modalities across all structures. For arytenoid cartilage and epiglottis invasion, MRI showed a non-significant trend towards higher sensitivity. Summary receiver operating characteristic (SROC) curves indicated superior overall diagnostic accuracy for MRI. In T-staging, MRI consistently demonstrated a lower rate of understaging compared to CT across five studies. Conclusions: MRI had superior diagnostic performance compared to CT in detecting laryngeal cartilage and deep space invasion; it had significantly higher sensitivity, comparable specificity, and lower risk of understaging, thus supporting the use of MRI for accurate pretreatment T-staging. However, given the limited number of studies, the results should be interpreted with caution, and further studies are needed to confirm our findings.

## Simple Summary

Laryngeal cancer is a serious disease affecting the voice box, and accurate staging before treatment is crucial for determining the best approach. Two main imaging techniques—computed tomography (CT scan) and magnetic resonance imaging (MRI)—are used to assess whether the cancer has invaded surrounding cartilage and tissues. However, it remains unclear which method is more reliable. This study systematically compared the diagnostic accuracy of CT and MRI in detecting cartilage invasion in laryngeal cancer patients. We found that MRI was significantly more sensitive than CT in detecting invasion of the thyroid cartilage, cricoid cartilage, and other important structures, while both methods showed similarly high specificity. MRI also provided more accurate tumor staging overall. These findings suggest that MRI should be considered the preferred imaging method for pre-treatment evaluation of laryngeal cancer, as it reduces the risk of missing hidden invasion and helps doctors make better treatment decisions, particularly when organ-preserving therapies are being considered.

## 1. Introduction

Laryngeal cancers are ranked twentieth among the most prevalent cancers worldwide [1]. They constitute a major burden, accounting for nearly a third of head and neck cancers [2]. The number of new cases reached more than 188 thousand in 2022, with more than 100,000 deaths [1]. Laryngeal cancer has several risk factors, including smoking, consuming alcohol, and gastroesophageal reflux [2]. The early detection of laryngeal cancer is essential to improve the prognosis of these patients since advanced stages are associated with higher mortality [3].

The management options for laryngeal cancer include surgery, radiotherapy, chemotherapy, or a combination of them [4,5]. The T staging of laryngeal cancer is important in determining the appropriate treatment approach. For instance, patients with advanced stages undergo surgical management, whereas patients with early stages undergo laryngeal preservation. The invasion of the laryngeal cartilage is categorized as T4a [4,5]. While histopathological examination is considered the gold standard for the detection of cartilage invasion, preoperative imaging remains essential for optimal treatment [5,6].

There are multiple imaging modalities for staging of laryngeal cancer, including computed tomography (CT) and magnetic resonance imaging (MRI). Currently, CT is the most commonly used imaging modality since it is readily available. In contrast, magnetic resonance imaging (MRI) offers high spatial resolution, especially among soft tissue structures; however, artifacts could arise during coughing or swallowing [7]. Nonetheless, there is no consensus on the optimal imaging modality. Although MRI is considered more accurate than CT, it could result in overstaging. Meanwhile, although CT is considered more specific, it could lead to understaging [8]. Determining the optimal imaging modality is important since inaccurate staging could affect disease recurrence and patients’ survival owing to a suboptimal treatment strategy [6].

While recent advancements in diagnostic accuracy using hyperspectral imaging and artificial intelligence-based radiomics have shown promising results, the current clinical practice relies on the traditional imaging techniques [9]. Several studies provided a head-to-head comparison between CT and MRI accuracy in staging of laryngeal cancer [8,10,11,12], and although several systematic reviews [13,14,15,16] were conducted, a direct head-to-head comparison of diagnostic performance between CT and MRI in patients with laryngeal cancer was not performed. Moreover, the performance of CT and MRI in detecting the invasion of each laryngeal cartilage, as well as surrounding structures such as the paraglottic space and anterior commissure, is yet to be determined.

Given these research gaps, we conducted this systematic review and meta-analysis to provide a direct head-to-head comparison of the diagnostic performance of CT and MRI in patients with laryngeal cancer. This synthesis of evidence is essential to guide current clinical practice and establish a benchmark against which emerging diagnostic technologies could be evaluated [9].

## 2. Materials and Methods

We conducted this systematic review and meta-analysis following the Preferred Reporting Items for Systematic Reviews and Meta-Analyses (PRISMA) [17]. The protocol was prospectively registered in PROSPERO (registration number: CRD420261279225).

### 2.1. Literature Search

We searched Web of Science, Scopus, and PubMed in November 2025 using the following keywords (“Magnetic Resonance Imaging” OR “MR Tomography” OR “MRI Scan” OR MRI) AND (“X-Ray Computed Tomography” OR “Computed X-Ray Tomography” OR “Xray Computed Tomography” OR “Computed tomography”) AND (((Cancer OR cancers OR neoplasm OR neoplasms OR carcinoma) AND (Larynx OR Laryngeal)) OR “Laryngeal Cancer” OR “laryngeal carcinoma”). The full search strategy is present in the Supplementary File.

### 2.2. Eligibility Criteria and Study Selection

We included cohort studies that included patients aged ≥ 18 with primary laryngeal cancer who underwent both CT and MRI as index tests and histopathological examination of the tumor as the reference test, where the diagnostic accuracy of both modalities was compared in detecting cancer staging or laryngeal invasion.

We excluded animal studies, studies not available in English, reviews, case reports, abstracts, and letters. Additionally, we excluded studies that did not provide separate data for patients with laryngeal cancer and studies that did not provide separate data for each modality. Also, the studies that included patients with recurrent cancer, patients receiving chemo or radiotherapy, or those including fewer than 10 patients were excluded. When studies with overlapping populations were found, we included the most recent and comprehensive one and excluded the other.

The results of the literature search were collected in an Excel sheet and screened through two phases. First, we conducted title and abstract screening of the retrieved studies, and then we screened the full text of the eligible studies.

### 2.3. Data Extraction and Quality Assessment

The authors conducted the data extraction using Excel sheets. The extracted data from the included studies were the general characteristics, baseline characteristics, and outcomes. The general characteristics of trials included the country, time of conduction, study design, and the sample size. The baseline characteristics of patients included age, gender, and the type of surgery. The outcomes involved the accuracy in the detection of invasion of laryngeal cartilages collected as true positive, true negative, false positive, and false negative values.

The quality of the included studies was evaluated using the QUADAS-2 tool [18]. The tool involves four domains, which are patient selection, index test, reference standard, and flow and timing. All the domains were assessed in terms of risk of bias, whereas the applicability was evaluated for the first three domains. Signaling questions for each domain were categorized as Yes, No, or unclear, and accordingly, the domains were judged as low risk, unclear, or high risk of bias.

### 2.4. Statistical Analysis

We performed the analysis using RevMan 5.4 software and R software (version 4.4.3). For the primary analysis, we performed a bivariate meta-analysis using a generalized linear mixed model (GLMM) to compare the sensitivity and specificity of CT and MRI. This accounts for between-study heterogeneity and the within-study correlation between sensitivity and specificity. We used the likelihood ratio tests to compare nested models and determine whether test accuracy differed significantly between CT and MRI. The summary estimates were back-transformed to the probability scale, and absolute and relative differences were calculated along with their corresponding 95% confidence intervals. The summary receiver operating characteristic (SROC) plots were generated using RevMan, using the parameters calculated from R. Additionally, we conducted a sensitivity analysis using the univariate random effects model when fewer than five studies were available for an outcome, as the univariate random-effects models could be more stable when limited data are available. The sensitivity and specificity were analyzed separately using generalized linear mixed-effects models with a binomial likelihood and a logit transformation in R. Furthermore, in the analysis of laryngeal cartilage invasion, we performed a sensitivity analysis by excluding the studies that used laser cordectomy since direct histological evaluation of laryngeal cartilage was not feasible. Studies with isolated zero cells (zero false positive, true positive, false negative, or true negative) were retained when model convergence was achieved. However, studies with multiple zero cells were excluded when model non-estimability or non-convergence occurred.

## 3. Results

### 3.1. Search Results and Study Selection

**Figure 1:**
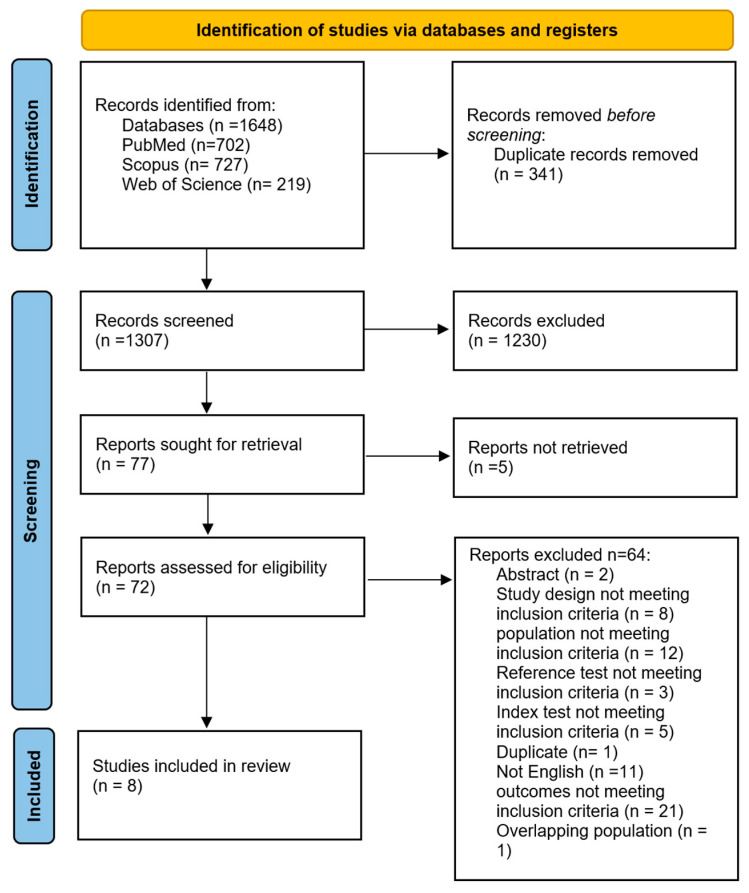
PRISMA Flow Chart Showing Identification and Selection of Included Studies.

The literature search retrieved 1648 articles, of which 341 were duplicates (Figure 1). Title and abstract screening was performed on 1307 articles, while 72 articles were screened according to their full text. Finally, eight studies [8,11,12,19,20,21,22,23] were included in this systematic review, of which six [8,12,19,20,21,23] were meta-analyzed.

### 3.2. Characteristics of Included Studies

The summary and baseline characteristics of the included studies are shown in Table 1 and Table 2. Six studies were prospective [8,12,19,20,21,23], while two were retrospective [11,22]. The sample size of the included studies ranged from 16 to 45. Among the included studies, the data for patients with CT and MRI were complete except for Kraft et al. [21], Pucetaite et al. [23], and Paone et al. [22], where the number of patients with available MRI data was fewer than CT. Furthermore, Pucetaite et al. conducted a per-case analysis, as more than one focus of cartilage could be investigated in the same patient [23]. The follow-up duration ranged from 12 to 72 weeks. The mean age of the participants ranged from 60 to 67.1, and the percentage of males ranged from 79.8% to 100%. The percentage of patients with squamous cell carcinoma ranged from 90% to 100%.

### 3.3. Quality Assessment

**Figure 2:**
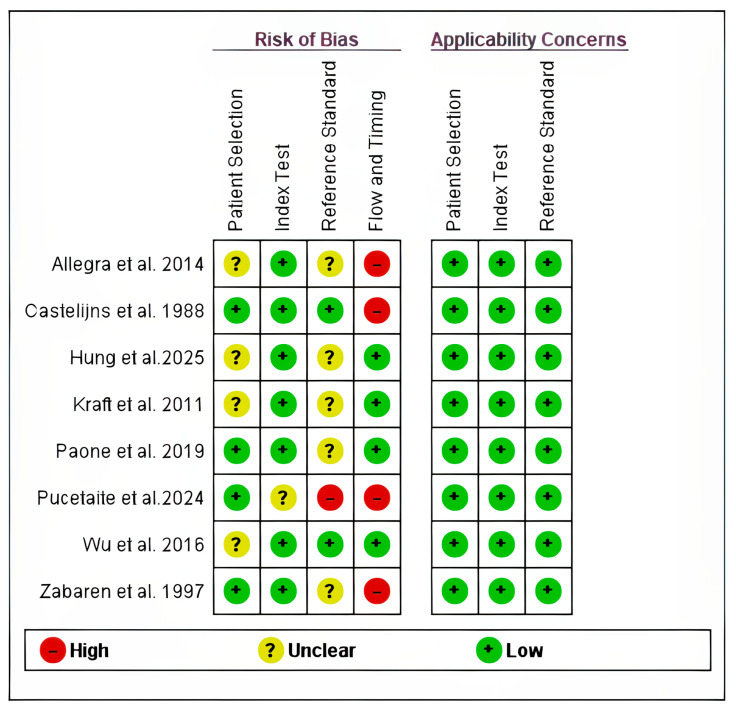
Risk of Bias and Applicability Concerns Summary for Included Studies [8,11,12,19,20,21,22,23].

**Figure 3:**
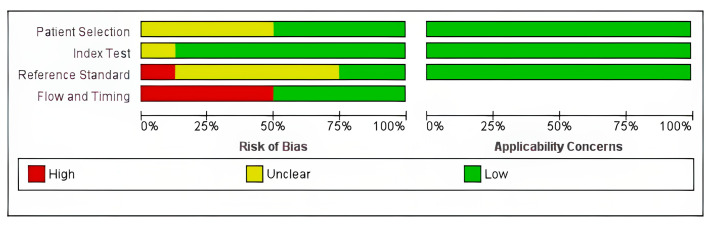
Risk of bias and applicability concerns summary graph.

The quality assessment summary and graph are shown in Figure 2 and Figure 3. All the included studies had low risk in the applicability domains. Four studies had unclear risk in the patients’ selection domain, as the study did not provide information on the inclusion of consecutive patients [8,11,19,21] while only one study had unclear risk in the index test domain [23], as the study did not provide information on whether the index test was interpreted without knowledge of the results of the reference standard. Five studies [8,12,19,21,22] had unclear risk in the reference test domain since there was no clear information on whether the reference tests were interpreted without knowledge of the index test results, while one study had high risk due to knowledge of the index test results [23]. Four studies had high risk in the flow and timing domain since not all the patients were included in the analysis [12,19,20,23].

### 3.4. Outcomes

#### 3.4.1. Thyroid Invasion

The pooled data from six studies [8,12,19,20,21,23] showed that CT had a sensitivity of 0.55 (95% CI 0.42–0.68) and a specificity of 0.96 (95% CI 0.79–0.99), while MRI had a sensitivity of 0.98 (95% CI 0.33–1.00) and a specificity of 0.86 (95% CI 0.64–0.95) in detecting thyroid cartilage invasion. CT was less sensitive than MRI, with an absolute sensitivity difference of −0.43 (95% CI −0.59 to −0.26) and a relative sensitivity of 0.56 (95% CI 0.44–0.73), while there was no significant difference in specificity, as shown in Table 3. The sensitivity analysis following exclusion of kraft et al., and allegra et al. that used laser cordectomy demonstrated similar findings in sensitivity and specificty, as shown in Supplementary Table S1 [19,21].

**Figure 4:**
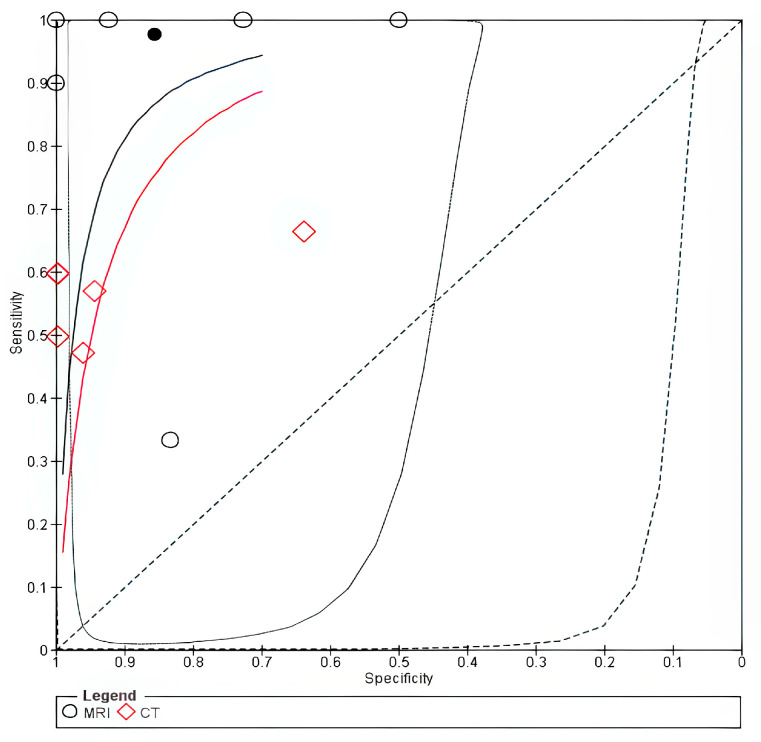
SROC of Thyroid invasion. The solid line represents the SROC curve for MRI, and the dashed line represents the SROC curve for CT. Circles (○) represent individual study estimates for MRI, and diamonds (◇) represent individual study estimates for CT. The filled circle (●) indicates the summary operating point for MRI, and the filled diamond indicates the summary operating point for CT.

The forest plot is shown in Supplementary Figure S1. The SROC curve for MRI was closer to the upper-left corner than that for CT, thus indicating superior overall diagnostic performance for thyroid cartilage invasion, as shown in Figure 4.

#### 3.4.2. Cricoid Cartilage Invasion

**Figure 5:**
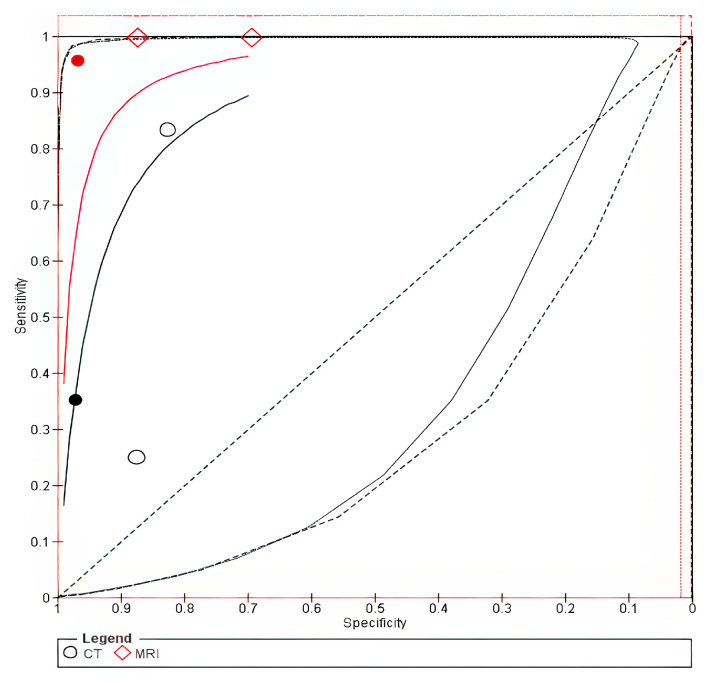
SROC of cricoid invasion. The solid line represents the SROC curve for MRI, and the dashed line represents the SROC curve for CT. Circles (○) represent individual study estimates for MRI, and diamonds (◇) represent individual study estimates for CT. The filled circle (●) and filled diamond (◆) indicate the summary operating points for MRI and CT, respectively.

The pooled data from four studies [8,12,19,20] showed that CT had a low sensitivity of 0.35 (95% CI 0.06–0.83) and a high specificity of 0.97 (95% CI 0.84–1.00), whereas MRI had a sensitivity of 1.00 (95% CI 0.00–1.00) with a specificity of 0.97 (95% CI 0.74–1.00) in detecting cricoid cartilage invasion. CT was less sensitive than MRI, with an absolute sensitivity difference of −0.65 (95% CI −1.14 to −0.15), while there was no significant difference in specificity, as shown in Table 3. The forest plot is shown in Supplementary Figure S2. The SROC curve for MRI lay closer to the upper-left corner, suggesting superior accuracy; however, this finding is based on a small number of studies and should be interpreted cautiously, as shown in Figure 5. The bivariate sensitivity analysis with exclusion of studies that used laser cordectomy showed similar findings, as shown in Supplementary Table S1. On the other hand, the univariate sensitivity analysis with and without exclusion of studies that used laser cordectomy showed no significant change in sensitivity, as shown in Supplementary Tables S1 and S2. However, this analysis was limited to two studies due to the exclusion of zero cells.

#### 3.4.3. Arytenoid Cartilage Invasion

The pooled data from five studies [8,12,19,20,21] showed that CT had a pooled sensitivity of 0.58 (95% CI 0.14–0.92) and a specificity of 0.96 (95% CI 0.77–0.99), whereas MRI showed a higher sensitivity of 0.81 (95% CI 0.58–0.93) with a similarly high specificity of 0.98 (95% CI 0.86–1.00). However, there was no significant difference between the two modalities in the sensitivity or specificity of detecting arytenoid cartilage invasion, as shown in Table 3. The forest plot is shown in Supplementary Figure S3.

**Figure 6:**
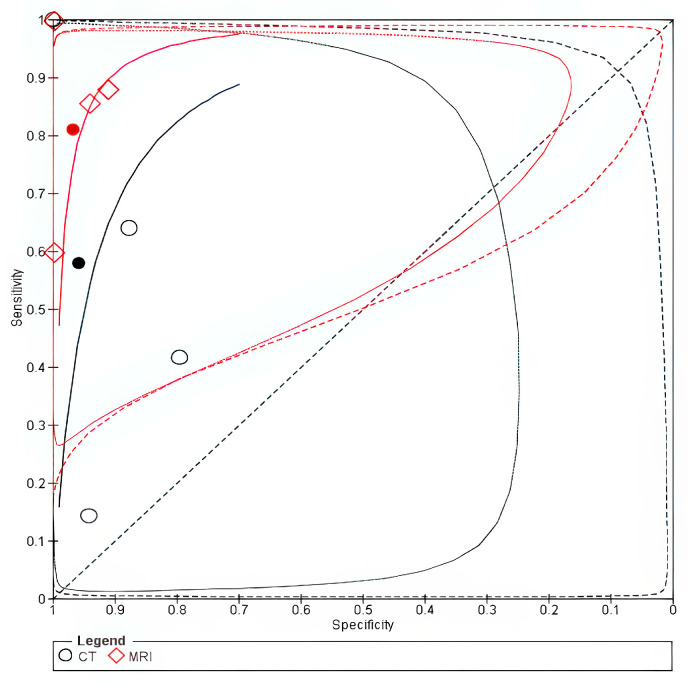
Arytenoid invasion. The solid line represents the SROC curve for MRI, and the dashed line represents the SROC curve for CT. Circles (○) represent individual study estimates for MRI, and diamonds (◇) represent individual study estimates for CT. The filled circle (●) and filled diamond (◆) indicate the summary operating points for MRI and CT, respectively.

The SROC curve for MRI lay closer to the upper-left corner, suggesting superior accuracy, as shown in Figure 6. The sensitivity analysis following exclusion of studies that used laser cordectomy showed no significant difference in specificity between both modalities, while there was a significant difference in sensitivity −0.63 [−1, −0.25] only in the bivariate model, as shown in Supplementary Table S1. However, it should be noted that the analysis was based on three studies in the bivariate and only two studies in the univariate model due to the exclusion of zero cells due to multiple zero cells and model non-estimability.

#### 3.4.4. Epiglottis Invasion

**Figure 7:**
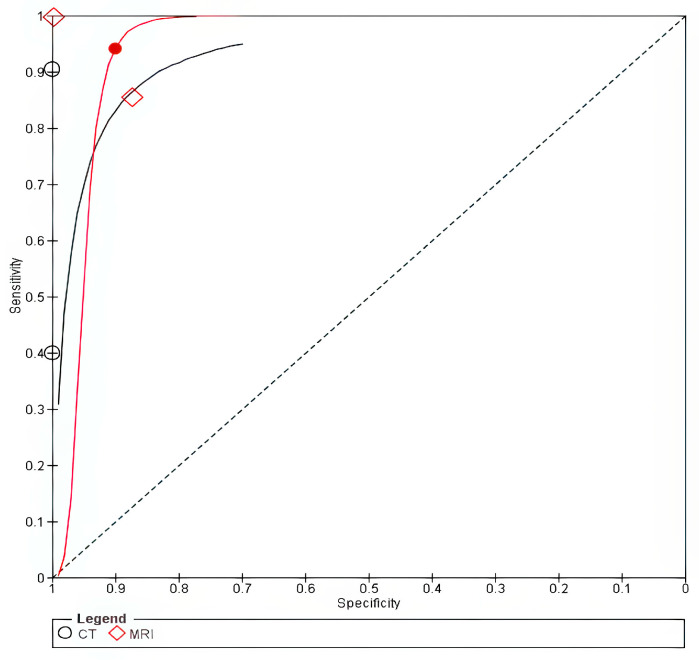
Epiglottitis invasion. The solid line represents the SROC curve for MRI, and the dashed line represents the SROC curve for CT. Circles (○) represent individual study estimates for MRI, and diamonds (◇) represent individual study estimates for CT. The filled circle (●) and filled diamond (◆) indicate the summary operating points for MRI and CT, respectively.

The pooled data from two studies [20,21] showed that CT had a sensitivity of 0.72 (95% CI 0.28–0.95) and a specificity of 1.00 (95% CI 0–1), whereas MRI showed a sensitivity of 0.94 (95% CI 0.68–0.99) and a specificity of 0.90 (95% CI 0.53–0.99). However, there was no significant difference between the two modalities in the sensitivity or specificity of detecting epiglottic invasion, as shown in Table 3. The forest plot is shown in Supplementary Figure S4. The univariate analyses showed comparable findings, as shown in Supplementary Table S2. The SROC curve for MRI lay closer to the upper-left corner, suggesting superior accuracy; however, this finding is based on a small number of studies and should be interpreted cautiously, as shown in Figure 7.

#### 3.4.5. Paraglottic Space Invasion

**Figure 8:**
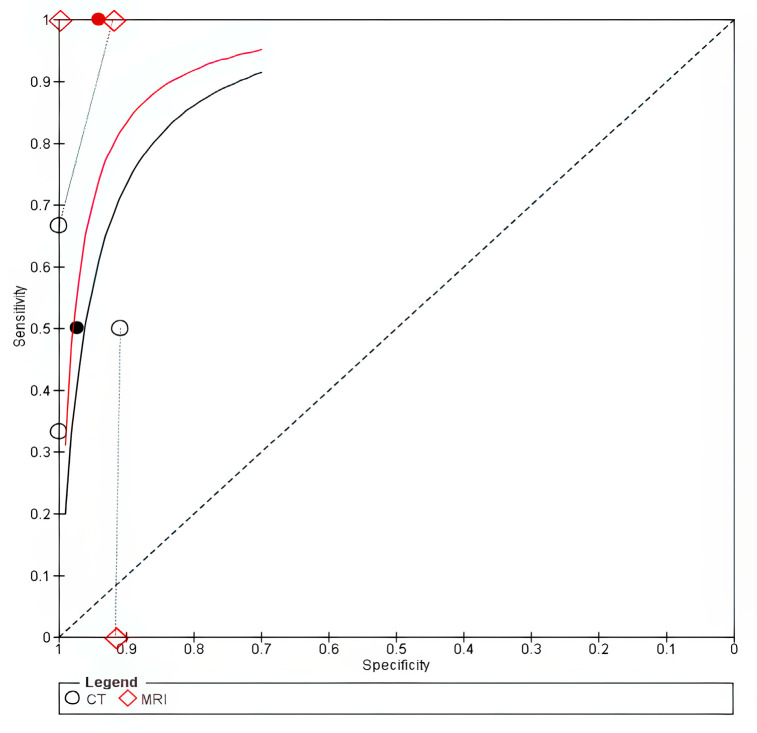
Paraglotic space. The solid line represents the SROC curve for MRI, and the dashed line represents the SROC curve for CT. Circles (○) represent individual study estimates for MRI, and diamonds (◇) represent individual study estimates for CT. The filled circle (●) and filled diamond (◆) indicate the summary operating points for MRI and CT, respectively.

The pooled data from three studies [8,19,21] showed that CT had a pooled sensitivity of 0.50 (95% CI 0.33–0.67) and a specificity of 0.97 (95% CI 0.87–0.99), whereas MRI showed a sensitivity of 1.00 (95% CI 0–1) and a specificity of 0.94 (95% CI 0.83–0.98). CT was less sensitive than MRI, with an absolute sensitivity difference of −0.5 (95% CI −0.68 to −0.32) and a relative difference of 0.5 (95% CI 0.35–0.72), while there was no significant difference in specificity, as shown in Table 3. The forest plot is shown in Supplementary Figure S5. However, the univariate sensitivity analysis showed no significant difference in pooled sensitivity, as shown in Supplementary Table S2. The SROC curve for MRI lay closer to the upper-left corner, suggesting superior accuracy; however, this finding is based on a small number of studies and should be interpreted cautiously, as shown in Figure 8.

#### 3.4.6. Anterior Commissure Invasion

**Figure 9:**
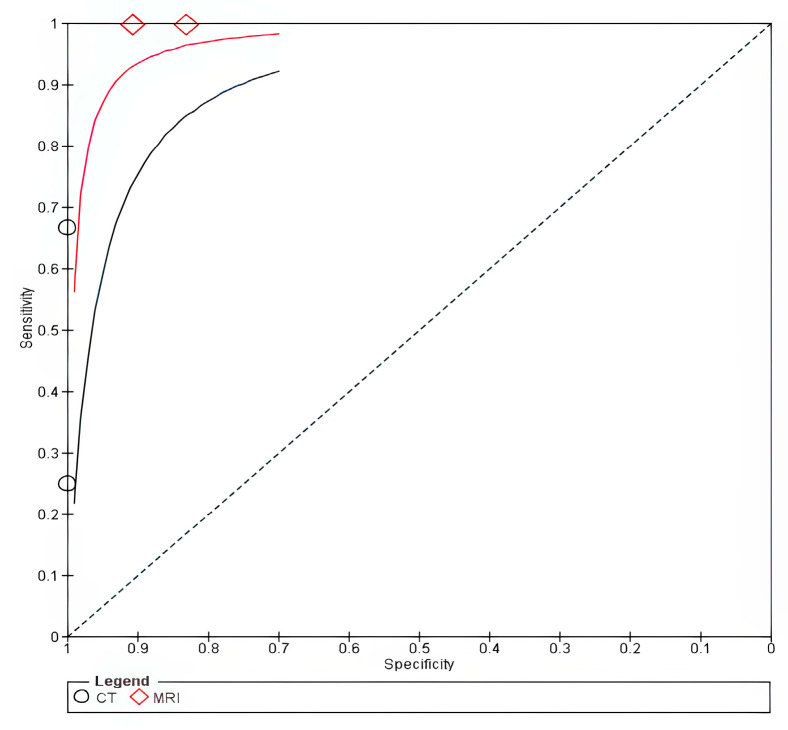
Anterior commissure, invasion. The solid line represents the SROC curve for MRI, and the dashed line represents the SROC curve for CT. Circles (○) represent individual study estimates for MRI, and diamonds (◇) represent individual study estimates for CT. The filled circle (●) and filled diamond (◆) indicate the summary operating points for MRI and CT, respectively.

The pooled data from two studies [8,19] showed that CT had a sensitivity of 0.46 (95% CI 0.21–0.74) and a specificity of 1 (95% CI 0–1), whereas MRI showed a sensitivity of 1 (95% CI 0–1) and a specificity of 0.88 (95% CI 0.73–0.96), as shown in Table 3. The forest plot is shown in Supplementary Figure S6. In the univariate random-effects sensitivity analysis, MRI was significantly more sensitive than CT, with an absolute sensitivity difference of 0.48 (95% CI 0.1 to 0.86), as shown in Supplementary Table S2. The SROC curve for MRI lay closer to the upper-left corner, suggesting superior accuracy; however, this finding is based on a small number of studies and should be interpreted cautiously, as shown in Figure 9.

#### 3.4.7. T Staging

Five studies [8,11,12,19,22] reported the performance of CT and MRI in overstaging and understaging of patients with laryngeal cancer. Allegrra et al. reported that MRI correctly staged 16 out of 20 patients, while four were overstaged, and CT correctly staged 14 out of 20 patients, where six patients were understaged. Hung et al. reported that MRI correctly staged 25 out of 31 patients, while four were overstaged and one was understaged; CT correctly staged 22 out of 31 patients, where eight patients were understaged, while one was overstaged. Paone et al. found that MRI correctly staged all 10 included patients, while CT correctly staged 20 out of 27 patients; six patients were understaged, while one was overstaged. Wu et al. showed that MRI correctly staged 23, while CT correctly staged 15 out of 26 patients. MRI understaged three patients, while CT overstaged two patients and understaged nine patients. Zabaren et al. found that MRI correctly staged 39, while CT correctly staged 36 out of 45 patients. MRI overstaged five patients and understaged one patient, while CT overstaged three patients and understaged six patients. A summary of the correct staging, understaging, and overstaging is present in Supplementary Table S3.

## 4. Discussion

Our systematic review and meta-analysis compared the diagnostic performance of CT and MRI in detecting cartilage invasion and cancer staging in patients with laryngeal cancer. We found that the sensitivity of MRI was significantly higher than that of CT in detecting thyroid cartilage invasion. Furthermore, MRI demonstrated higher sensitivity in detecting the invasion of cricoid cartilage, paraglottic space, and the anterior commissure. However, there was no significant difference between CT and MRI in the sensitivity of detecting arytenoid cartilage or epiglottis invasion. Moreover, there was no significant difference in specificity between both modalities in thyroid cartilages, paraglottic space, or anterior commissure invasion. The included studies showed that MRI was more accurate than CT in T staging of laryngeal cancer; MRI tended to overstage while CT tended to understage patients.

Accurate staging of laryngeal cancer is essential since overstaging could result in aggressive surgical management leading to a deterioration in functional outcomes such as speech or swallowing and a low quality of life. On the other hand, understaging could lead to inadequate management and increased risk of mortality [24,25]. Kim et al. found that one in every four patients with laryngeal cancer had a false diagnosis [26]. In our study, we found that MRI had an overall higher accuracy, as demonstrated in the SROC curves. This was mainly driven by the increased sensitivity, as no significant difference in specificity was detected. However, MRI tended to overstage patients while CT tended to understage patients. This could be explained as MRI tends to overestimate cancer boundaries because of the inflammation surrounding the tumor [27]. In contrast, Egmond et al. found that MRI overstaged 6% and under-staged 13% of patients with laryngeal cancer [28]. However, this could be attributed to different criteria, as they only included patients with glottic cancer stages I and II. Thus, patients with stage I or II who were overstaged by MRI to stage III or IV were not included.

We found that MRI had a higher sensitivity compared to CT in the detection of thyroid cartilage invasion, while there was no significant difference in specificity. Similarly to these findings, Castelijns et al. found similar specificity; however, Zabaren et al. reported that MRI was more sensitive but significantly less specific than CT [12,20]. This could be explained by several factors. For instance, different MRI techniques such as the DWI could increase its specificity, owing to a better differentiation between tumor margins and the surrounding edema [27]. Indeed, several techniques could improve the accuracy of traditional imaging modalities. The DWI technique, for example, could improve the accuracy of MRI and reduce its overstaging tendency in patients with laryngeal cancer [29,30]. However, since only four of our included studies used DWI, we recommend that further studies should investigate the impact of using DWI in patients with laryngeal cancer. Furthermore, using artificial intelligence-based radiomics could improve the accuracy of imaging modalities in cancer detection; however, they are not yet integrated in routine clinical care, given the existing limitations, such as their limited generalizability [31]. Thus, further research could improve their real-world applicability.

The original site of the laryngeal cancer could impact MRI accuracy. Hintz et al. showed that MRI had lower specificity in detecting cartilage invasion when the tumor was of glottic region compared to non-glottic origin [32]. Among our included studies, there was a high incidence of glottic primary site. This is consistent with the higher prevalence in males [33], which is typical of laryngeal cancer. Similarly, Hartl et al. found that the specificity of CT in the detection of thyroid cartilage invasion was affected by the tumor site; it was reduced when the tumor involved the anterior commissure [34]. However, considering the limited data, subgroup analyses investigating these factors were not conducted in our study.

Similarly to the findings of Hintz et al., Lim et al. found that the diagnostic performance of imaging techniques varied according to the subsite. For instance, the anterior commissure was the most affected site, having a low specificity (42%) and the highest number of false positive findings [35]. This is clinically relevant since after involvement of the anterior commissure, the tumor may easily spread to nearby structures such as the subglottic region or even outside the larynx [24,36]. Furthermore, the anterior commissure involvement is a prognostic factor for cancer recurrence [37,38]. Hung et al. found that CT was more specific (100% for CT vs. 91% for MRI) and less sensitive (67% for CT vs. 100% for MRI) in detecting anterior commissure invasion than MRI [8]. In our study, we found that MRI had a significantly higher sensitivity estimate than CT, while no significant difference in specificity was observed.

While several meta-analyses investigated the diagnostic performance of CT and MRI, they did not investigate whether a difference in diagnostic performance existed between both modalities in each cartilage. For instance, Wang et al. investigated only the diagnostic performance of MRI in laryngeal cartilage invasion [14]. However, they did not specify the investigated cartilages. Meanwhile, Cho et al. investigated the diagnostic performance of CT and MRI in patients with laryngeal and hypopharyngeal cancer [13]. However, they did not provide a head-to-head comparison. Furthermore, they only reported the diagnostic performance for thyroid, cricoid, and arytenoid cartilages for the subgroup of patients who received CT only. Whereas we compared the diagnostic performance of both modalities in different laryngeal cartilages. We found no significant difference in sensitivity and specificity between the two modalities in the detection of epiglottis or arytenoid cartilage invasion. Similarly, Kraft et al. found no significant difference in the diagnostic performance between both modalities in epiglottic invasion [21].

In our study, we found that MRI significantly had higher sensitivity compared to CT in the detection of cricoid cartilage and paraglotic space invasion. However, owing to the limited number of studies and bivariate model instability and singular fits, we conducted an exploratory analysis using the univariate model. However, there was no significant difference in sensitivity. Thus, these findings should be interpreted with caution, and further studies are needed to confirm the significantly superior accuracy of MRI compared to CT. Moreover, among our included studies, only Hung et al. investigated the accuracy of combining CT and MRI compared to MRI only. They found that using both techniques had comparable accuracy to using MRI only; it did not reduce the overstaging tendency of MRI. However, since this was investigated by only one study, further studies are needed to investigate the impact of using combined CT and MRI in patients with laryngeal cancer.

Strengths and Limitations

Our study has several strengths. First, to our knowledge, this is the first systematic review and meta-analysis to provide a head-to-head comparison of the diagnostic performance of CT and MRI in detecting cartilage invasion in patients with laryngeal cancer. Moreover, we investigated the diagnostic performance in each cartilage type in contrast to the previous meta-analysis. Moreover, we excluded overlapped populations in contrast to the meta-analysis by Wu et al. [11], which included both Zabaren et al. and Becker et al. [10,12]. Additionally, we investigated the effect of both index tests on understaging and overstaging of patients with laryngeal cancer. In contrast to other meta-analyses that included both laryngeal and hypopharyngeal cancer, we restricted our inclusion criteria to laryngeal cancer only, since, as stated before, the performance of imaging modality could be affected by the original tumor site. Moreover, the included studies were conducted in various settings and most of them were prospectively conducted.

Our study has some limitations. First, the number of included studies was limited, and the sample size of the included studies was small. Second, some of the included studies had unclear or high risk of bias in some of the domains. Third, there was heterogeneity in the imaging procedures, such as MRI field strengths, contrast utilization, and reader expertise, as well as stages of cancer. Owing to inconsistent reporting and the limited number of studies, protocol-level subgroup analyses were not feasible. Thus, further studies with standardized imaging protocols are needed. Moreover, there were insufficient data for comparing different histological types of laryngeal cancer. However, all the included studies included 90% or more of patients with SCC. Thus, our findings might not apply to patients with different histological subtypes. We excluded patients who underwent radiotherapy and those with recurrent cancer. However, this could be justified as they might develop post-radiation changes, including developing chondronecrosis, which simulates laryngeal cancer, resulting in false positive results. Whereas, patients who undergo previous resection of the tumor have distorted anatomy, which might affect the diagnostic performance of both modalities [5,24]. We restricted the reference standard to histopathological examination in patients undergoing surgery. This might have introduced a selection bias, since not all patients with laryngeal cancer in real-world data would undergo laryngectomy. However, this resulted in reliable findings compared to other reported reference tests, such as clinical staging; Zabaren et al. found that clinical staging alone had an accuracy of only 55% [12]. Nonetheless, most of the included studies did not provide details on blinding of the pathologist; thus, they were judged as having unclear risk of bias in this domain. Some of the included studies used laser cordectomy since laryngeal cancer was at early stages; thus, cartilage invasion was pathologically assessed only in specimens containing perichondrium or cartilage. Thus, in cases where cartilage was not present, invasion was recorded as not identified, while tumors with suspected cartilage invasion did not undergo laser cordectomy but were treated with partial or total laryngectomy, allowing histological confirmation. Therefore, laser cordectomy cases contributed to true-negative assessments. Nonetheless, we performed a sensitivity analysis by excluding these studies and found similar findings. Furthermore, there were some limitations in the analysis. The extremely wide confidence interval (0–1) reflected substantial uncertainty related to sparse data. Due to the small number of included studies, estimation of the bivariate random-effects model resulted in boundary (singular) fits. Results from the bivariate model are therefore interpreted cautiously, with emphasis that SROC curves should be interpreted as descriptive due to the limited number of studies. We conducted a sensitivity analysis using the univariate random effects model, since univariate random-effects models may be more stable when very few studies are available, and could provide adequate estimates as stated by Takwoingi et al. [39]. However, since they ignore the correlation between sensitivity and specificity, they were considered only as exploratory sensitivity analyses. Since both models yielded inconsistent results in some of the investigated outcomes, these results should be interpreted cautiously, and further studies are needed to confirm our findings.

Clinical implications

This systematic review and meta-analysis provided evidence on the diagnostic performance of CT and MRI in laryngeal cancer staging and detecting laryngeal cartilage invasion. We found that MRI had higher sensitivity in detecting thyroid cartilage invasion. Moreover, MRI had higher sensitivity in detecting the invasion of cricoid cartilage, paraglottic space, and the anterior commissure. There was no significant difference in specificity between the two modalities. Thus, MRI should be considered to detect cartilage invasion since its higher sensitivity reduces the risk of missing occult invasion. Although MRI was more accurate in T staging, it was more likely to overstage, while CT was more likely to understage patients. MRI overstaging of tumor extent could potentially influence clinicians toward more aggressive treatment, such as total laryngectomy, in cases where organ-preserving strategies might be considered. Meanwhile, CT understaging of tumor spread could lead to inappropriate selection of conservative treatments. Thus, MRI could be used to provide a more reliable therapeutic decision, particularly where organ-preservation strategies are considered, since understaging could affect patients’ survival. Nonetheless, CT has an important role in clinical practice, considering its high specificity, wider availability, and lower cost. It remains valuable for initial evaluation, assessment of patients with contraindications to MRI, or motion artifact. However, given the limitations of the analysis and the limited number of included studies, the review findings should be interpreted cautiously, and further studies are needed to confirm our findings.

## 5. Conclusions

Our study showed that MRI is superior to CT in the preoperative assessment of laryngeal cancer owing to its significantly higher sensitivity for detecting invasion of laryngeal structures. MRI had higher sensitivity compared to CT for detecting the invasion of thyroid cartilage, cricoid cartilage, paraglottic space, and anterior commissure. However, there was no significant difference in sensitivity between both modalities in detection of arytenoid cartilage and epiglottis invasion. Meanwhile, both modalities had comparable high specificity. Overall, MRI was more accurate than CT in T-staging, although it tends to overstage patients, while CT tends to understage them. However, given the limited number of included studies, these findings should be interpreted with caution. Further studies with head-to-head comparisons are needed to confirm our findings. The effect of the origin of the primary tumor and its histological subtype on the diagnostic performance of imaging modalities needs further investigation. Studies should also assess the impact of new imaging techniques, such as DWI and radiomics, as well as the impact of using multiple imaging techniques.

## Figures and Tables

**Table 1:** Study Characteristics and Imaging Specifications of Included Studies in the Meta-Analysis.

Study ID	Year	Country	Time of Study Conduction	Study Design	Sample	Population	CT Modality Type	CT Contrast	MRI Modality type	MRI Contrst	DWI
Allegra et al. [19]	2014	Italy	Aug 2011–Nov 2013	PC	26	Suspected Glottic Laryngeal Cancer	Toshiba Aquilion CX 64-Slice	Y	Philips Achieva 1.5 T	NR	Y
Castelijns et al. [20]	1988	Netherlands	NR	PC	16	Untreated laryngeal/hypopharyngeal cancer	Tomoscan 350 (Philips)	NR	Teslacon I 0.6 T (Technicare)	NR	NR
Hung et al. [8]	2025	Vietnam	Jan 2022–Nov 2024	PC	31	Early-stage glottic cancer	GE Revolution 128-slice	Y	1.5 T MRI	Y	Y
Kraft et al. [21]	2011	Germany	NR	PC	76	Laryngeal cancer undergoing microlaryngoscopy	NR	NR	NR	NR	NR
Paone et al. [22]	2019	Switzerland	Jan 2010–Dec 2016	RC	27	Advanced glottic carcinoma	NR	Y	NR	Y	NR
Pučetaitė et al. [23]	2024	Lithuania	2021–2023	PC	27	Histopathologically proven laryngeal SCC	TSX-301 (Toshiba)	Y	Philips Ingenia 3.0 T	Y	Y
Wu et al. [11]	2016	China	May 2012–Jan 2014	RC	26	Laryngeal SCC with AVC involvement	Toshiba 64-slice	Y	3.0 T MRI	Y	Y
Žaberen et al. [12]	1997	Switzerland	Oct 1992–Mar 1996	PC	45	Surgically treated laryngeal neoplasms	Somatom Plus (Siemens)	NR	GE 1.5 T Perf. Plus	Y	NR

Abbreviations: PC = Prospective Cohort; RC = Retrospective Cohort; CE = Contrast-Enhanced; DWI = Diffusion-Weighted Imaging; Y = Yes; NR = Not Reported; N = Sample Size; AVC = Anterior Vocal Commissure; SCC = Squamous Cell Carcinoma.

**Table 2:** Patient Demographics, Surgical Approaches, and Tumor Characteristics of Included Studies.

Study	Age, Years Mean (SD)	Male n (%)	Surgical Procedures	Tumor Location	Histology
Allegra et al. 2014 [19]	63.6 (6.8)	26 (100)	SCPL-CHEP: 10 (38.5%) TL: 4 (15.4%) CO2 laser cordectomy: 4 (15.4%) SCPL + laser cordectomy: 2 (7.7%)	Glottic: 26 (100%)	SCC: 18 (90%)
Castelijns et al. 1988 [20]	52–76 *	15 (93.8)	TL: 13 (81.3%) SGL: 3 (18.8%)	Supraglottic: 4 (25%) Glottic: 8 (50%)	SCC: 16 (100%)
Hung et al. 2025 [8]	62.4 (7.0)	31 (100)	SCPL-CHEP: 11 (35.5%) TL: 5 (16.1%) TC surgery + VCR: 3 (9.7%)	NR	SCC: 30 (96.8%)
Kraft et al. 2011 [21]	63.0 (8.2)	71 (93)	Cordectomy, laser resection, partial or total laryngectomy	Glottic: 27 (36%) Supraglottic: 15 (20%) Glotto-supraglottic: 13 (17%) Glotto-subglottic: 10 (13%) Transglottic: 11 (14%)	SCC: 73 (96%) Myxoid sarcoma: 1 Fibrous histiocytoma: 1 Neuroendocrine SCC: 1
Paone et al. 2019 [22]	67.1 (6.3)	24 (88.9)	TL ± thyroidectomy	Glottic: 27 (100%)	SCC: 27 (100%)
Pučetaitė et al. 2024 [23]	63.0 (8.7)	27 (100)	NR	Glottic: 14 (51.9%) Transglottic: 13 (48.1%)	SCC: 27 (100%)
Wu et al. 2016 [11]	61.5 (8.8)	26 (100)	TL: 8 (30.8%) FPL: 8 (30.8%) SCPL: 10 (38.5%)	NR	SCC: 26 (100%)
Žaberen et al. 1997 [12]	60.0 (10.8)	44 (79.8)	TL: 41 (91.1%) SGL: 3 (6.7%) Subtotal laryngectomy: 1 (2.2%)	Supraglottic: 7 (15.6%) Subglottic: 1 (2.2%) Glottic-supraglottic: 5 (11.1%) Glottic-subglottic: 15 (33.3%) Transglottic: 17 (37.7%)	SCC: 43 (95.6%) Undifferentiated NPC: 1 (2.2%) Adenocarcinoma: 1 (2.2%)

Abbreviations: SCPL-CHEP, Supracricoid partial laryngectomy with cricohyoidoepiglottopexy; TL, Total laryngectomy; SGL, Supraglottic laryngectomy; FPL, Frontal partial laryngectomy; TC, Thyroid cartilage; VCR, Vocal cord resection; SCC, Squamous cell carcinoma; NPC, Nasopharyngeal carcinoma; NR, Not reported; * Age range provided instead of mean (SD).

**Table 3:** Diagnostic Performance of CT versus MRI in Detecting Invasion of Laryngeal Structures.

Detection of Invasion of	No. of Studies	CT		MRI		Absolute Difference (Between CT and MRI)		Relative Difference (Between CT and MRI)	
		Sensitivity [95%CI]	Specificity [95%CI]	Sensitivity [95%CI]	Specificity [95%CI]	Sensitivity [95%CI]	Specificity [95%CI]	Sensitivity [95%CI]	Specificity [95%CI]
Thyroid	6	0.55 [0.42, 0.68]	0.96 [0.79, 0.99]	0.98 [0.33, 1]	0.86 [0.64, 0.95]	−0.43 [−0.59, −0.26]	0.11 [−0.06, 0.27]	0.56 [0.44, 0.73]	1.13 [0.93, 1.36]
Cricoid	4	0.35 [0.06, 0.83]	0.97 [0.84, 1]	1 [0, 1]	0.97 [0.74, 1]	−0.65 [−1.14,−0.15]	0.004 [−0.09, 0.09]	0.35 [0.09, 1.44]	1 [0.91, 1.1]
Arytenoid	5	0.58 [0.14, 0.92]	0.96 [0.77, 0.99]	0.81 [0.58, 0.93]	0.98 [0.86, 1]	−0.23 [−0.77, 0.31]	−0.025 [−0.11, 0.06]	0.72 [0.29, 1.78]	0.97 [0.89, 1.07]
Epiglottis	2	0.72 [0.28, 0.95]	1 [0, 1]	0.94 [0.68, 0.99]	0.9 [0.53, 0.99]	−0.22 [−0.62, 0.18]	0.1 [−0.09, 0.29]	0.77 [0.45, 1.31]	1.1 [0.9, 1.37]
Paraglottic space	3	0.5 [0.33, 0.67]	0.97 [0.87, 0.99]	1 [0, 1]	0.94 [0.83, 0.98]	−0.5 [−0.68, −0.32]	0.03 [−0.05, 0.11]	0.5 [0.35, 0.72]	1.03 [0.95, 1.12]
Anterior commissure	2	0.46 [0.21, 0.74]	1 [0, 1]	1 [0, 1]	0.88 [0.73, 0.96]	−0.54 [NaN, NaN]	0.12 [NaN, NaN]	0.46 [NaN, NaN]	1.13 [NaN, NaN]

## Data Availability

The original contributions presented in this study are included in the article/Supplementary Material. Further inquiries can be directed to the corresponding author(s).

## Supplementary Materials

The following supporting information can be downloaded at: https://www.mdpi.com/article/10.3390/cancers18040583/s1. Table S1: Sensitivity analysis excluding studies that useed laser cordectomy; Table S2: Sensitivity analysis using the univariate random effects; Supplementary Table S3: Summary of correct staging, overstaging, and understaging; Figure S1: Forest plot showing sensitivity and specificity of CT and MRI in detecting thyroid cartilage invasion; Figure S2. Forest plot showing sensitivity and specificity of CT and MRI in detecting cricoid cartilage invasion; Figure S3: Forest plot showing sensitivity and specificity of CT and MRI in detecting arytenoid cartilage invasion; Figure S4 Forest plot showing sensitivity and specificity of CT and MRI in detecting epiglottis invasion; Figure S5: Forest plot showing sensitivity and specificity of CT and MRI in detecting paraglottic space invasion; Figure S6. Forest plot showing sensitivity and specificity of CT and MRI in detecting anterior commissure invasion.
