# Supplementary material for: Comparison of Diagnostic Performance Between CT and MRI for Detection of Cartilage Invasion and Tumor Staging in Patients with Laryngeal Cancer: A Systematic Review and Meta-Analysis
Source: Cancers (Basel). 2026 Feb 10;18(4):583. doi: 10.3390/cancers18040583 (PMC12938627; doi:10.3390/cancers18040583)
Supplement: Supplementary file 1 [file cancers-18-00583-s001.zip › cancers-4119805-supplementary.pdf]

Search strategy:-

Search date: 28/11/2025

<u>PubMed:</u>

("Magnetic Resonance Imaging"[Mesh] OR "Magnetic Resonance Imaging" OR “NMR Imaging” OR Zeugmatography OR “Steady-State Free Precession MRI” OR “Steady State Free Precession MRI” OR “NMR Tomography” OR “MR Tomography” OR “Proton Spin Tomography” OR “Magnetization Transfer Contrast Imaging” OR fMRI OR “Functional MRI” OR “Functional MRIs” OR “Functional Magnetic Resonance Imaging” OR “MRI Scan” OR “MRI Scans” OR “Chemical Shift Imagings” OR “Chemical Shift Imaging” OR “Spin Echo Imaging” OR “Spin Echo Imagings” OR “Magnetic Resonance Image” OR “Magnetic Resonance Images” OR MRI) AND ("Tomography, X-Ray Computed"[Mesh] OR "X-Ray Computed Tomography” OR Tomodensitometry OR “Computed X-Ray Tomography” OR “Xray Computed Tomography” OR “Xray Computed Tomographies” OR “X-Ray CAT Scan” OR “X-Ray CAT Scan” OR “Transmission Computed Tomography” OR “X-Ray CT Scan” OR “X-Ray CT Scans” OR “X-Ray Computerized Tomography” OR “X Ray Computerized Tomography” OR “Computed X Ray Tomography” OR “CT X Ray” OR “CT X Rays” OR “X-Ray Computer Assisted Tomography” OR “X Ray Computer Assisted Tomography” OR “Cine-CT” OR “Cine CT” OR “Electron Beam Computed Tomography” OR “Electron Beam Tomography” OR “X-Ray Computerized Axial Tomography” OR “X Ray Computerized Axial Tomography” OR “Computed tomography”) AND (((Cancer OR cancers OR neoplasm OR neoplasms OR carcinoma) AND (Larynx OR Laryngeal)) OR "Laryngeal Neoplasms"[Mesh] OR "Laryngeal Neoplasms" OR “Larynx Neoplasm” OR “Larynx Neoplasms” OR “Cancer of Larynx” OR “Larynx Cancers” OR “Cancer of the Larynx” OR “Laryngeal Cancer” OR “Laryngeal Cancers” OR “ Larynx Cancer” OR “laryngeal carcinoma”)

Results: 702

Search fields: All fields

<u>WOS:</u>

("Magnetic Resonance Imaging" OR “NMR Imaging” OR Zeugmatography OR “Steady-State Free Precession MRI” OR “Steady State Free Precession MRI” OR “NMR Tomography” OR “MR Tomography” OR “Proton Spin Tomography” OR “Magnetization Transfer Contrast Imaging” OR fMRI OR “Functional MRI” OR “Functional MRIs” OR “Functional Magnetic Resonance Imaging” OR “MRI Scan” OR “MRI Scans” OR “Chemical Shift Imagings” OR “Chemical Shift Imaging” OR “Spin Echo Imaging” OR “Spin Echo Imagings” OR “Magnetic Resonance Image” OR “Magnetic Resonance Images” OR MRI) AND ("X-Ray Computed Tomography” OR Tomodensitometry OR “Computed X-Ray Tomography” OR “Xray Computed Tomography” OR “Xray Computed Tomographies” OR “X-Ray CAT Scan” OR “X-Ray CAT Scan” OR “Transmission Computed Tomography” OR “X-Ray CT Scan” OR “X-Ray CT Scans” OR “X-Ray Computerized Tomography” OR “X Ray Computerized Tomography” OR “Computed X Ray Tomography” OR “CT X Ray” OR “CT X Rays” OR “X-Ray Computer Assisted Tomography” OR “X Ray Computer Assisted Tomography” OR “Cine-CT” OR “Cine CT” OR “Electron Beam Computed Tomography” OR “Electron Beam Tomography” OR “X-Ray Computerized Axial Tomography” OR “X Ray Computerized Axial Tomography” OR “Computed tomography”) AND (((Cancer OR cancers OR neoplasm OR neoplasms OR

carcinoma) AND (Larynx OR Laryngeal)) OR "Laryngeal Neoplasms" OR “Larynx Neoplasm” OR “Larynx Neoplasms” OR “Cancer of Larynx” OR “Larynx Cancers” OR “Cancer of the Larynx” OR “Laryngeal Cancer” OR “Laryngeal Cancers” OR “ Larynx Cancer” OR “laryngeal carcinoma”)

Results: 219
Search fields: Topic

<u>Scopus :</u>

("Magnetic Resonance Imaging" OR “NMR Imaging” OR Zeugmatography OR “Steady-State Free Precession MRI” OR “Steady State Free Precession MRI” OR “NMR Tomography” OR “MR Tomography” OR “Proton Spin Tomography” OR “Magnetization Transfer Contrast Imaging” OR fMRI OR “Functional MRI” OR “Functional MRIs” OR “Functional Magnetic Resonance Imaging” OR “MRI Scan” OR “MRI Scans” OR “Chemical Shift Imagings” OR “Chemical Shift Imaging” OR “Spin Echo Imaging” OR “Spin Echo Imagings” OR “Magnetic Resonance Image” OR “Magnetic Resonance Images” OR MRI) AND ("X-Ray Computed Tomography” OR Tomodensitometry OR “Computed X-Ray Tomography” OR “Xray Computed Tomography” OR “Xray Computed Tomographies” OR “X-Ray CAT Scan” OR “X-Ray CAT Scan” OR “Transmission Computed Tomography” OR “X-Ray CT Scan” OR “X-Ray CT Scans” OR “X-Ray Computerized Tomography” OR “X Ray Computerized Tomography” OR “Computed X Ray Tomography” OR “CT X Ray” OR “CT X Rays” OR “X-Ray Computer Assisted Tomography” OR “X Ray Computer Assisted Tomography” OR “Cine-CT” OR “Cine CT” OR “Electron Beam Computed Tomography” OR “Electron Beam Tomography” OR “X-Ray Computerized Axial Tomography” OR “X Ray Computerized Axial Tomography” OR “Computed tomography”) AND (((Cancer OR cancers OR neoplasm OR neoplasms OR carcinoma) AND (Larynx OR Laryngeal)) OR "Laryngeal Neoplasms" OR “Larynx Neoplasm” OR “Larynx Neoplasms” OR “Cancer of Larynx” OR “Larynx Cancers” OR “Cancer of the Larynx” OR “Laryngeal Cancer” OR “Laryngeal Cancers” OR “ Larynx Cancer” OR “laryngeal carcinoma”)

Results: 727
Search fields: : TITLE-ABS-KEY

Supplementary Table S1. Sensitivity analysis excluding studies that useed laser cordectomy.

**Detection of invasion of**	**no. Of studies**	**Analysis model**	**CT**		**MRI**		**Absolute difference between CT and MRI (CT-MRI)**		**Relative difference between CT and MRI (CT/MRI)**	
			**sensitivity [95%CI]**	**Specificity [95%CI]**	**sensitivity [95%CI]**	**Specificity [95%CI]**	**sensitivity [95%CI]**	**Specificity [95%CI]**	**sensitivity [95%CI]**	**Specificity [95%CI]**
**Thyroid**	**4**	**Bivariate**	**0.55 [0.39, 0.70]**	**0.95 [0.61, 1]**	**0.98 [0.73, 1]**	**0.79 [0.52, 0.93]**	**-0.42 [-0.6, -0.25]**	**0.16 [-0.07, 0.4]**	**0.57 [0.42, 0.76]**	**1.21 [0.91, 1.6]**

		univariate	0.55 [0.4, 0.7]	0.96 [0.6, 1]	0.97 [0.83, 1]	0.77 [0.52, 0.92]	-0.38 [-0.2, -0.56]	0.13 [-0.16 , 0.42]	0.59 [0.44 , 0.80]	1.17 [0.81 , 1.7]
**Cricoid**	**3**	**Bivariate**	**0.46 [0.09, 0.88]**	**0.95 [0.77, 0.99]**	**1 [0, 1]**	**0.93 [0.61, 0.99]**	**-0.54 [-1.07, -0.02]**	**0.02 [-0.14, 0.19]**	**0.46 [0.14, 1.45]**	**1.02 [0.86, 1.22]**
		**univariate**	**0.64 [0.19, 0.93]**	**0.94 [0.72, 0.99]**	**1 [0, 1]**	**0.93 [0.55, 0.99]**	**-0.32 [- 0.84, 0.19]**	**0.04 [- 0.19 , 0.27]**	**0.66 [0.29 , 1.49]**	**1.05 [0.81 , 1.36]**
**Arytenoids**	**3**	**Bivariate**	**0.25 [0.045, 0.69]**	**0.97 [0.85, 1]**	**0.87 [0.71, 0.95]**	**0.97 [0.87, 0.99]**	**-0.63 [-1, -0.25]**	**0.004 [-0.06, 0.07]**	**0.28[0.06, 1.22]**	**1 [0.94, 1.08]**
		**univariate**	**0.4197 [0.1169 ; 0.7980]**	**0.97 [0.79, 1]**	**0.88 [0.71, 0.95]**	**0.97 [0.85, 0.99]**	**-0.42 [- 0.86, 0.009]**	**-0.01 [- 0.12, 0.09]**	**0.52 [0.21 , 1.26]**	**0.99 [0.88 , 1.1]**

Supplementary Table S2. Sensitivity analysis using the univariate random effects

**Sensitivity analysis (univariate random effects meta-analysis)**									
**Detection of invasion of**	**no. Of studies**	**CT**		**MRI**		**Absolute difference (between MRI and C)**		**Relative difference (between MRI and C)**	
		**sensitivity (95%CI)**	**Specificity (95%CI)**	**sensitivity (95%CI)**	**Specificity (95%CI)**	**sensitivity (95%CI)**	**Specificity (95%CI)**	**sensitivity (95%CI)**	**Specificity (95%CI)**
**Cricoid**	4	0.64 [0.19, 0.93]	0.97 [0.75, 1]	1 [0, 1]	0.97 [0.62, 1]	0.36 [- 0.09, 0.82]	0.001 [-0.18, 0.12]	1.57[0.51, 4.87]	1.002[0.88, 1.14]
**Epiglottis**	2	0.72 [0.27, 0.95]	1.0000 [0, 1.0000]	0.94 [0.69, 0.99]	0.9 [0.53, 0.99]	0.20 [-0.28, 0.69]	-0.09 [-0.31, 0.14]	1.29 [0.44, 2.14]	0.91 [0.67, 1.14]
**paraglottic space**	3	0.50 [0.33, 0.67]	0.97 [0.70, 1]	1 [0.18, 1]	0.94 [0.83, 0.98]	0.25 [-0.11, 0.62]	-0.6 [-10.1 , 9]	1.51 [0.68, 2.34]	0.99 [0.89, 1.10]
**Anterior commissure**	2	0.46 [0.20, 0.75]	1.0000 [0, 1]	1 [0, 1]	0.88 [0.73, 0.96]	0.48 [0.1, 0.86]	-0.09 [-0.219, 0.032]	2.03 [0.42, 3.65]	0.90 [0.78, 1.03]

Supplementary Table S3. Summary of correct staging, overstaging, and understaging

**Study ID**	**Index test**	**T staging**			
		**correct staging**	**overstaged**	**understaged**	**sample**
**Allegra et al. 2014 [1]**	CT	14	0	6	20
	MRI	16	4	0	20
**Hung et al.2025 [2]**	CT	22	1	8	31
	MRI	25	5	1	31
**Paone et al. 2019 [3]**	CT	20	1	6	27
	MRI	10	0	0	10
**Wu et al. 2016 [4]**	CT	15	2	9	26
	MRI	23	0	3	26
**Zabaren et al. 1997 [5]**	CT	36	3	6	45
	MRI	39	5	1	45

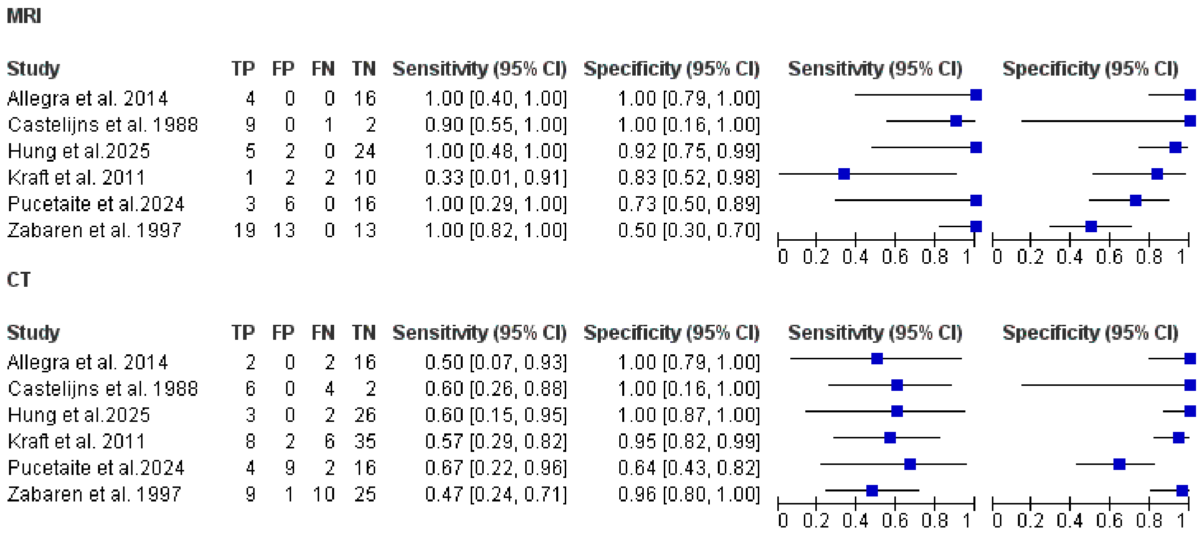

Supplementary Figure S1. Forest plot showing sensitivity and specificity of CT and MRI in detecting thyroid cartilage invasion [1,2,5–8].

CT

Study	TP	FP	FN	TN	Sensitivity (95% CI)	Specificity (95% CI)
Allegra et al. 2014	0	0	0	20	Not estimable	1.00 [0.83, 1.00]
Castelijns et al. 1988	1	1	3	7	0.25 [0.01, 0.81]	0.88 [0.47, 1.00]
Hung et al.2025	0	0	0	31	Not estimable	1.00 [0.89, 1.00]
Zabaren et al. 1997	15	4	3	19	0.83 [0.59, 0.96]	0.83 [0.61, 0.95]

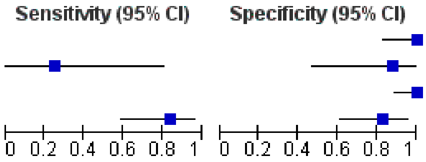

MRI

Study	TP	FP	FN	TN	Sensitivity (95% CI)	Specificity (95% CI)
Allegra et al. 2014	0	0	0	20	Not estimable	1.00 [0.83, 1.00]
Castelijns et al. 1988	4	1	0	7	1.00 [0.40, 1.00]	0.88 [0.47, 1.00]
Hung et al.2025	0	0	0	31	Not estimable	1.00 [0.89, 1.00]
Zabaren et al. 1997	18	7	0	16	1.00 [0.81, 1.00]	0.70 [0.47, 0.87]

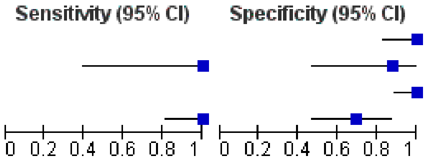

Supplementary Figure S2. Forest plot showing sensitivity and specificity of CT and MRI in detecting cricoid cartilage invasion [1,2,5,6].

CT

Study	TP	FP	FN	TN	Sensitivity (95% CI)	Specificity (95% CI)
Allegra et al. 2014	2	0	0	18	1.00 [0.16, 1.00]	1.00 [0.81, 1.00]
Castelijns et al. 1988	1	1	6	16	0.14 [0.00, 0.58]	0.94 [0.71, 1.00]
Hung et al.2025	0	0	0	62	Not estimable	1.00 [0.94, 1.00]
Kraft et al. 2011	5	8	7	31	0.42 [0.15, 0.72]	0.79 [0.64, 0.91]
Zabaren et al. 1997	16	7	9	50	0.64 [0.43, 0.82]	0.88 [0.76, 0.95]

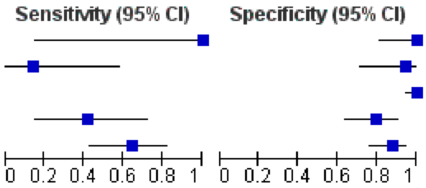

MRI

Study	TP	FP	FN	TN	Sensitivity (95% CI)	Specificity (95% CI)
Allegra et al. 2014	2	0	0	18	1.00 [0.16, 1.00]	1.00 [0.81, 1.00]
Castelijns et al. 1988	6	1	1	16	0.86 [0.42, 1.00]	0.94 [0.71, 1.00]
Hung et al.2025	0	0	0	62	Not estimable	1.00 [0.94, 1.00]
Kraft et al. 2011	3	0	2	10	0.60 [0.15, 0.95]	1.00 [0.69, 1.00]
Zabaren et al. 1997	22	5	3	52	0.88 [0.69, 0.97]	0.91 [0.81, 0.97]

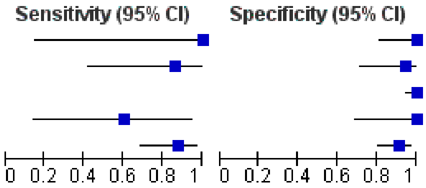

Supplementary Figure S3. Forest plot showing sensitivity and specificity of CT and MRI in detecting arytenoid cartilage invasion [1,2,5-7].

CT

Study	TP	FP	FN	TN	Sensitivity (95% CI)	Specificity (95% CI)
Castelijns et al. 1988	4	0	6	2	0.40 [0.12, 0.74]	1.00 [0.16, 1.00]
Kraft et al. 2011	19	0	2	30	0.90 [0.70, 0.99]	1.00 [0.88, 1.00]

MRI

Study	TP	FP	FN	TN	Sensitivity (95% CI)	Specificity (95% CI)
Castelijns et al. 1988	10	0	0	2	1.00 [0.69, 1.00]	1.00 [0.16, 1.00]
Kraft et al. 2011	6	1	1	7	0.86 [0.42, 1.00]	0.88 [0.47, 1.00]

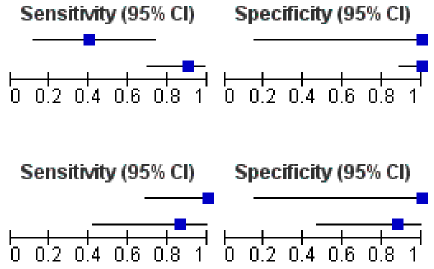

Supplementary Figure S4. Forest plot showing sensitivity and specificity of CT and MRI in detecting epiglottis invasion [6,7].

CT

Study	TP	FP	FN	TN	Sensitivity (95% CI)	Specificity (95% CI)
Allegra et al. 2014	2	0	4	14	0.33 [0.04, 0.78]	1.00 [0.77, 1.00]
Hung et al.2025	4	0	2	25	0.67 [0.22, 0.96]	1.00 [0.86, 1.00]
Kraft et al. 2011	9	3	9	30	0.50 [0.26, 0.74]	0.91 [0.76, 0.98]

MRI

Study	TP	FP	FN	TN	Sensitivity (95% CI)	Specificity (95% CI)
Allegra et al. 2014	6	0	0	14	1.00 [0.54, 1.00]	1.00 [0.77, 1.00]
Hung et al.2025	6	2	0	23	1.00 [0.54, 1.00]	0.92 [0.74, 0.99]
Kraft et al. 2011	0	1	3	11	0.00 [0.00, 0.71]	0.92 [0.62, 1.00]

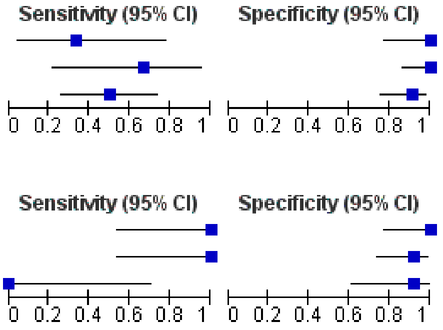

Supplementary Figure S5. Forest plot showing sensitivity and specificity of CT and MRI in detecting paraglottic space invasion [1,2,7].

CT

Study	TP	FP	FN	TN	Sensitivity (95% CI)	Specificity (95% CI)
Allegra et al. 2014	2	0	6	12	0.25 [0.03, 0.65]	1.00 [0.74, 1.00]
Hung et al.2025	6	0	3	22	0.67 [0.30, 0.93]	1.00 [0.85, 1.00]

MRI

Study	TP	FP	FN	TN	Sensitivity (95% CI)	Specificity (95% CI)
Allegra et al. 2014	8	2	0	10	1.00 [0.63, 1.00]	0.83 [0.52, 0.98]
Hung et al.2025	9	2	0	20	1.00 [0.66, 1.00]	0.91 [0.71, 0.99]

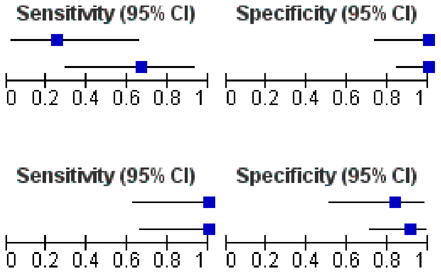

Supplementary Figure S6. Forest plot showing sensitivity and specificity of CT and MRI in detecting anterior commissure invasion [1,2].

References

1. Allegra, E.; Ferrise, P.; Trapasso, S.; Trapuzzano, O.; Barca, A.; Tamburrini, S.; Garozzo, A. Early glottic cancer: Role of mri in the preoperative staging. *BioMed Res. Int.* **2014**, *2014*, 890385. https://doi.org/10.1155/2014/890385.
2. Hung, N.D.; Lan, D.T.P.; Trang, B.H.; Van-Tuan, N.; Huong, N.D.; Duc, N.M. The role of 1.5 tesla mri and mdct in the preoperative staging of early-stage glottic cancer. Russ. Electron. J. Radiol. 2025, 15, 53–64. https://doi.org/10.21569/2222-7415-2025-15-2-53-64.
3. Paone, G.; Martucci, F.; Espeli, V.; Ceriani, L.; Treglia, G.; Ruberto, T.; Richetti, A.; Piantanida, R.; Giovanella, L.; Zannetti, A. F-FDG-PET/CT Imaging in Advanced Glottic Cancer: A Tool for Clinical Decision in Comparison with Conventional Imaging. Contrast Media Mol. Imaging 2019, 2019, 4051206. https://doi.org/10.1155/2019/4051206.
4. Wu, J.H.; Zhao, J.; Li, Z.H.; Yang, W.Q.; Liu, Q.H.; Yang, Z.Y.; Liao, B.; Li, X.L.; Wang, B.; Qin, H.; et al. Comparison of CT and MRI in Diagnosis of Laryngeal Carcinoma with Anterior Vocal Commissure Involvement. *Sci. Rep.* **2016**, *6*, 30353. https://doi.org/10.1038/srep30353.
5. Zbären, P.; Becker, M.; Läng, H. Staging of laryngeal cancer: Endoscopy, computed tomography and magnetic resonance versus histopathology. In European Archives of Oto-Rhino-Laryngology; Springer: Berlin/Heidelberg, Germany, 1997; pp. S117–S122.
6. Castelijns, J.A.; Gerritsen, G.J.; Kaiser, M.C.; Valk, J.; van Zanten, T.E.G.; Golding, R.G.; Meÿer, C.J.L.M.; van Hattum, L.H.; Sprenger, M.; Bezemer, P.D.; et al. Invasion of laryngeal cartilage by cancer: Comparison of CT and MR imaging. RADIOLOGY 1988, 167, 199–206. https://doi.org/10.1148/radiology.167.1.3347723.
7. Kraft, M.; Bruns, N.; Hügens-Penzel, M.; Arens, C. Clinical value of endosonography in the assessment of laryngeal cancer. Head Neck 2013, 35, 195–200. https://doi.org/10.1002/hed.22949.
8. Pucėtaitė, M.; Farina, D.; Ryškienė, S.; Mitraitė, D.; Tarasevičius, R.; Lukoševičius, S.; Padervinskis, E.; Vaitkus, S. The Diagnostic Value of CEUS in Assessing Non-Ossified Thyroid Cartilage Invasion in Patients with Laryngeal Squamous Cell Carcinoma. J. Clin. Med. 2024, 13, 891. https://doi.org/10.3390/jcm13030891.
